# Co-morbidity of attention deficit hyperactivity disorder among children with seizure disorders at University of Gondar referral hospital Ethiopia (2016)

**DOI:** 10.1038/s41598-021-95751-8

**Published:** 2021-08-12

**Authors:** Haregewoin Mulat, Niguse Yegezaw, Tewodros Eyasu

**Affiliations:** 1 Department of Psychiatry, College of Medicine and Health Sciences, The University of Gondar, Gondar, Ethiopia; 2 College of Medicine and Health Sciences, Student’s Clinic, The University of Gondar, Gondar, Ethiopia

**Keywords:** Psychology, Medical research

## Abstract

Attention deficit hyperactivity disorder is a disorder in which a person is unable to control behavior due to difficulty in processing neural stimuli, accompanied by an extremely high level of motor activity. The prevalence is much higher ranging from 8 to 77% among children with seizure disorders than in the general population. When attention deficit hyperactivity disorder presents in children with seizure disorder, it makes the treatment complicated and the prognosis poor. Hence, understanding the magnitude of attention deficit hyperactivity disorder and associated factors would be important to have a policy intention towards these people and to design appropriate interventions. Therefore, the current study was conducted to determine the comorbidity of attention deficit hyperactivity disorder and associated factors in children with seizure disorders. A hospital-based cross-sectional study was conducted by taking 260 children who have follow ups in the pediatric seizure clinic. The systematic random sampling technique was used to recruit participants. A structured, pretested and interviewer-administered questionnaire which included questions on associated factors and standard disruptive behavioral disorder rating scale was used to collect data. Data were coded, entered and cleaned by using the Epi-Data version 3.1 and exported to SPSS version 20 for further analysis. The multivariate binary logistic regression was used to check the association between independent and dependent variables. Variables with significant associations were identified based on adjusted odds ratio, with a 95% CI and p-value of < 0.05 will be considered as statistically significant. The prevalence of attention deficit hyperactivity disorder among epileptic children was found to be 115 (44.2%),with a confidence interval of (38.1–50.5),out of which only 3 (2.6%) were detected as having mental health problems by the clinician. The predominant subtype was inattentive type 96 (61.1%). Factors significantly associated with attention deficit hyperactivity disorder were male sex (AOR = 2.70 CI 1.46–4.97), family history of seizure disorder (AOR = 2.42 CI 1.26–4.65), family history of mental illnesses (AOR = 4.14 CI 1.76–9.68), sudden onset of the seizure (AOR = 2.37 CI 1.32–4.27), and uncontrolled seizure (AOR = 2.55 CI 1.41–4.61). Attention deficit hyperactivity disorder was common among children with seizure disorders in the study area. Male sex, sudden onsets of seizure, family history of seizure, and that of other psychiatric disorders as well as uncontrolled seizures were factors that increased the odds of attention deficit hyperactivity disorder. Therefore, interventions that would address such factors would help to overcome further complications.

## Introduction

Attention deficit hyperactivity disorder (ADHD) is a disorder in which a person is unable to control behavior due to difficulty in processing neural stimuli, accompanied by an extremely high level of motor activity. It is one of the most common mental disorders that develop in children and becomes apparent in preschool and early school years. It is characterized by pervasive and impairing symptoms of inattention, hyperactivity, and impulsiveness that occur before 7 years of age^1^.

Symptoms of ADHD may increase risk-taking behavior. Consequently, the quality of life of individuals with ADHD may be impaired. ADHD can also be associated with a substantial economic burden for the individual, their family and societal healthcare services.

The prevalence of attention deficit hyper activity disorder (ADHD) in the general population ranges between 3 and 5%^2^. The prevalence is much higher ranging from 8 to 77% among children with epilepsy than in the general population^3^. The prevalence of ADHD among children with epilepsy in high-income countries ranges from 23 to 70%^4,5^. Clinical studies show that when ADHD presents in children with seizure disorders, it exerts its effect on the treatment and prognosis of the disorder. It can also have negative impacts on the affected child’s behavioral, learning, and social development. The studies suggest that 30–40% of children with epilepsy also present with ADHD^6,7^. Despite this high prevalence, ADHD often remains unrecognized and left without treatment.

Studies identified that the magnitude of the problem in children with epilepsy varied across the globe such as, 6.9% in Korea^8^, 42.2% in China^9^, 27.7% in Israel^10^, 29.1% in Brazil^11^, 12% in UK^12^, 28% in Norway^13^, 26.4% in USA^14^ and 60.4% in Iran^15^.

Another study conducted in central China showed that the magnitude of ADHD among children with epilepsy was 24.7%^16^.

A recent study in the Indian tertiary medical center revealed that the comorbidity of ADHD among epileptic children was 23%^17^.

According to a study in Tanzania, the comorbidity of ADHD among children with epilepsy showed that attention problem were more prevalent in children with epilepsy 53% compared with control groups (19%)^18^.

Previous studies revealed that age, sex, type of epilepsy syndrome^6,16^, earlier epilepsy onset, a longer period of antiepileptic medication, epileptic children’s receiving a combination of antiepileptic drugs^7^, and the frequency of seizures^15^ were the most common associated factors of ADHD among epileptic children.

Many of the studies were outside Africa, and the magnitude of ADHD among epileptic children in Ethiopia, particularly in the study area was poorly understood. Understanding the magnitude of ADHD and associated factors would be important to formulate policies and design appropriate interventions. Hence, this study aimed to assess the prevalence of comorbidity of ADHD and associated factors among epileptic children who have follow-ups at University of Gondar referral hospital’s pediatric seizure clinic.

## Methods

### Study setting, study period and design

A hospital-based cross-sectional design was used to assess ADHD and associated factors among children with seizure disorders attending the pediatric seizure clinic of the University of Gondar referral hospital. Gondar has located about 723 km from Addis Ababa and 65 km north of Lake Tana. The University of Gondar referral hospital provides tertiary care to the population of Gondar and its neighboring regions. The data were collected from September 13 to November 25, 2016.

#### Sample size determination and sampling technique

All Children attending the pediatric seizure clinic and aged between 6 and 17 years were taken as the source population while children who were available during data collection were considered as the study population. As per the information obtained from the pediatric seizure clinic, about 40 epileptic children with mothers or caregivers used to visit the clinic per week. The single population proportion formula with an assumption of 95% confidence level, a 5% margin of error and 50% prevalence and a 10% non-response rate was taken to determine the sample size as there were no similar studies in our country. Because the total population was less than 10,000, the correction formula was used to get the final sample size of 265.

### Data collection method and instrument

Data were collected from parents or caregivers by using the interview technique with the standard DBD rating scale for investigating the presence of ADHD. The scale consists of 45 items representing symptoms of Disruptive Behavior Disorders that are conduct disorder (CD), oppositional defiant disorder (ODD) and ADHD. Of the 45 items, only 18 were used in this study.

Children whose responses to six of the nine questions on hyper activity were “pretty much” or “very much” were considered as positive for the ADHD hyperactive subtype, while children whose responses to the same number of inattentive questions were “pretty much” or “very much” were considered as positive for the ADHD inattentive sub type. A structured questionnaire was developed to identify factors associated with ADHD, and patient charts were revised to get seizure related factors. The internal validity of the instrument was also checked (Cronbach’s alpha = 0.957) and found to be acceptable.

### Data quality assurance and analysis

The collected data were coded, entered and cleaned by using Epi-Data version 3.1 and exported to SPSS version 20. Frequencies and cross tabulations were used to summarize descriptive statistics of the data and tables and graphs were used for presentation. Bivariate logistic regression was first fitted to identify potential confounding factors and variables with p-values less than 0.2 were entered in to the multiple logistic regression model using the backward selection method to identify factors associated with depression. The adjusted odds ratio with a 95% confidence interval was calculated to report the strength and significance of the association.

Data quality was managed by training and appropriate supervision of data collectors by the principal investigator. A pre-test was done on 42 children at Feleg-Hiwot hospital pediatric seizure clinic, Bahirdar. During analyzing the pre-test The Cronbach’s Alpha for the pretest was 0.631 and Appropriate modifications were made on parts of the questionnaire which ask about family related factors and seizure related variables before the actual data collection.

### Ethical considerations

Ethical clearance was obtained from the University of Gondar Ethical Review committee (IRB). A written support letter was secured from the hospital chief executive officer and medical director. The benefit of the study for their children and the possible risks of participating in the study were discussed with parents of caregivers. Also the right to refuse and discontinue the interview at any time was kept. Informed written consent was sought from participant’s parents or caregivers before starting the data collection. Parents of children confirmed to have ADHD were advised and linked to the psychiatry clinic of the hospital.

All methods were performed following relevant guidelines and regulations of scientific studies.

## Result

### Socio-demographic and family related factors

A total of 260 children on follow up at Gondar University hospital pediatric seizure clinic participated in the study with a response rate of 98.1%. Out of the total participants, 158 (60.8%) were male sex, with a mean age of 10.5 ± 2.7 years (Table 1).

**Table 1:** Socio-demographic and family-related characteristics of participants (n = 260), University of Gondar referral hospital.

	Frequency	Percent (%)
**Age**		
7–11	184	70.8 mean age
≥ 12	76	29.2 10.5 ± 2.7 years
**Sex**		
Male	158	60.8
Female	102	39.2
**Religion**		
Orthodox	214	82.3
Others	46	17.7
**Ethnicity**		
Amhara	244	93.8
Others	16	6.2
**Is the mother alive**		
Yes	239	91.9
No	21	8.1
**Is the father alive**		
Yes	237	91.2
No	23	8.8
**Living arrangement**		
With both parents	222	85.4
One parent or others	38	14.6
**Known seizure disorder in the family**		
Yes	69	26.5
No	191	73.5
**Known mental illness in the family**		
Yes	41	15.8
No	219	84.2

### Pregnancy, delivery and childhood related factors

One fourth, 69 (26.5%), and 41 (15.8%) of the children had a family history of seizure disorder and psychiatric illness, respectively, while 32 (12.3%) had severe medical illness before the age of 7 years (Table 2).

**Table 2:** Pregnancy, delivery and childhood related characteristics of participants (n = 260), University of Gondar referral hospital.

	Frequency	Percent (%)
**Mother’s health during pregnancy**		
Healthy	251	96.5
Acute illness	3	1.5
Chronic illness	6	2.3
**Substance abuse during pregnancy**		
Yes	256	98.5
No	4	1.5
**Place of delivery**		
Home	190	73.1
Health institution	70	26.9
**Mode of delivery**		
SVD	219	84.2
Instrumental delivery	28	10.8
CS	13	5
**Birth complication**		
Yes	26	90
No	234	10
**Feeding**		
Exclusive breastfeeding	244	93.8
Formula	3	1.2
Mixed	13	5
**Health problem before the age of 7 years**		
Yes	228	87.7
No	32	12.3

### Seizure related factors

Most, 224 (86.2%) of the children were diagnosed with a generalized tonic–clonic seizure; among 135 (51.9%) participants’ seizure started suddenly. More than half 173 (66.5%) of the children had a seizure at least once per month before they were started medication. The seizure was not controlled among more than half 142 (54.6%) of the participants (Table 3).

**Table 3:** Seizure related factors (n = 260), University of Gondar referral hospital.

	Frequency	Percent (%)
**Age of set**		
Before age 7	152	58.5
Age 7 above	108	41.5
**One set**		
Sudden	135	51.9
Gradual	125	48.1
**Seizure type**		
GTC	224	86.2
Others	36	13.8
**Last seizure**		
Within 6 months	142	54.6
Before 6 months	118	45.4
**Number of medication**		
Single	181	69.6
Combination	79	30.4

### Prevalence of ADHD

**Figure 1:**
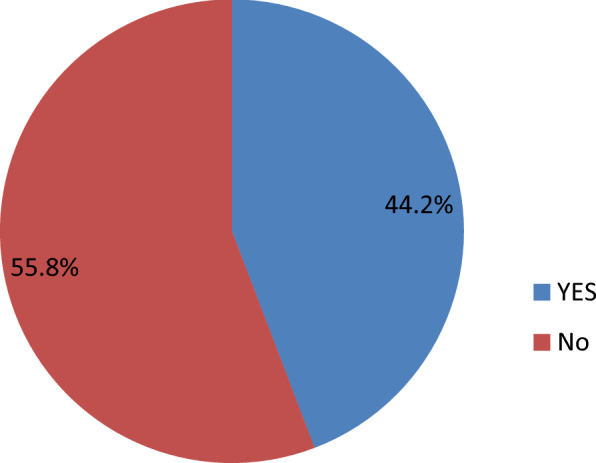
Magnitude of attention deficit hyperactivity disorder (ADHD).

As shown in Fig. 1, the magnitude of ADHD among epileptic children was 115 (44.2) with a 95% confidence interval of (38.1–50.5), out of which only 3 (2.6%) were detected as having mental health problems by the clinician. The predominant 96 (61.1%), subtype was inattentive type which is followed by the hyperactive subtype 61 (38.9%).

### Factors associated with ADHD

Among all the covariate age groups, sex, presence of mothers and fathers, living arrangements, family history of seizure, and mental illness, seizure type, onset and status were found to have a p-value less than 0.2 in the bi-variable logistic regression and considered for the multiple logistic regression model. The model goodness of fit was tested using the Hosmer and Lemeshow test and the p-value was found to be 0.719 and revealed the model was good.

Factors significantly associated with ADHD were male sex, (AOR = 2.70 CI 1.46–4.97), family history of seizure disorder (AOR = 2.42 CI 1.26–4.65), family history of other mental illnesses (AOR = 4.14 (1.76–9.68), sudden onsets of seizure (AOR = 2.37 (1.32–4.27) and uncontrolled seizures, 2.55 (1.41–4.61) (Table 4).

**Table 4:** Factors associated with ADHD among children with seizure disorders at University of Gondar referral hospital.

	ADHD		COR (95% CI)	AOR (95% CI)
	Yes	No		
**Age**				
7–11	86	98	1.42 (0.82–2.45)	1.57 (0.82–3.02)
≥ 12	29	47	1.00	1.00
**Sex**				
Male	86	72	3.01 (1.76–5.11)	2.70 (1.46–4.97)*
Female	29	73	1.00	1.00
**Mother alive**				
Yes	100	3.41 (1.54–4.82)	0.659 (0.16–2.17)	
No	15	1.00	1.00	
**Father alive**				
Yes	98	139	4.02 (1.53–10.5)	0.38 (0.87–1.65)
No	17	6	1.00	1.00
**Living arrangement**				
Both parents	90	25	2.82 (1.37–5.81)	1.14 (0.38–3.61)
One parent or others	25	13	1.00	1.00
**Family Hx of seizure**				
Yes	43	26	2.73 (1.55–4.82)	2.42 (1.26–4.65)*
No	72	119	1.00	1.00
**Family Hx of mental illness**				
Yes	31	10	4.98 (2.32–10.68)	4.14 (1.76–9.68)*
No	84	135	1.00	1.00
**Seizure type**				
GTC	107	117	3.2 (1.39–7.33)	1.86 (0.73–4.75)
Others	8	28	1.00	1.00
**Seizure onset**				
Sudden	74	61	2.48 (1.50–4.11)	2.37 (1.32–4.27)*
Gradual	41	84	1.00	1.00
**Seizure status**				
Uncontrolled	75	67	2.18 (1.32–3.61)	2.55 (1.41–4.61)*
Controlled	118	78	1.00	1.00

*means P-value < 0.05.

## Discussion

Knowing the prevalence of ADHD among children with seizure disorders is vital to set policies and strategies for early identification and management of the disorder and to improve the prognosis of seizure disorder also the quality of life of children with seizure disorders.

The prevalence of ADHD among children with seizure disorder was 44.2% (95% CI 38.1%, 50.5%) which is lower than those of previous studies conducted in Iran in which the prevalence of ADHD was 60.4^15^ and Tanzania which showed that the prevalence is 53%^18^, the discrepancy will be due to sample size and assessment tool differences. The study conducted in Iran used a highly sensitive child symptom inventory. And also a study done in Tanzania took a large sample and used a highly sensitive child behavioral checklist The result of the current study is in line with the result of a study conducted in China in which the prevalence was 42.2%^9^. The current estimate is by far higher than the results of the studies conducted in Korea (6.9%)^8^, Israel (27.7%)^10^, Brazil (29.1%)^11^, Norway (28%)^13^, USA (26.4%)^14^, Central China (24.7%)^16^ and India (23%)^17^. The differences were due to variations in sample sizes, study populations and screening tools. The sample sizes in the other studies were lower than that of the current study. The studies conducted in Korea and India used the DSM-IV criterion which was a diagnostic manual, and epileptic children were observed to confirm the presence of ADHD symptoms. In addition, they excluded children who had an intellectual disability, other psychiatric disorders and chronic medical illnesses. In the USA, only children with new onsets of epilepsy diagnosed in the past 12 months were taken.

The current study was identified different factors that had associations with ADHD during the course of seizure disorders. Male sex was one of the associated factors of the magnitude of ADHD, a result in line with that of a study conducted in India^17^. This was different from what was reported from Korea^8^ and the USA^14^.

The other factor that had a significant association with ADHD was family history of seizure disorder. If a child had a family history of seizure disorders, the illness would be very severe, and the severity of the seizure disorder might increase the risk of developing childhood psychiatric disorders, including ADHD. Previous studies we reviewed did not assess family history of seizure disorder as an associated factor.

In contrast to a study in India^17^, family history of psychiatric illness was significantly associated with the magnitude of ADHD among children with seizure disorders. That was evidenced by the role of genetic predisposition for psychiatric disorders. Other studies did not assess family history of psychiatric disorders as a contributing factor.

The other contributing factor was an uncontrolled seizure. Children who had seizure episodes during the last 6 months, had more than two times increased risk for ADHD compared with children who were seizure-free for more than 6 months, similar to the finding in India^17^. It was however different from what were reported in Korea^8^ and USA^14^ which showed that children’s seizure status had no a significant associations with the prevalence of ADHD.

Sudden onset of seizure disorder is among the associated factors of the magnitude of seizure disorders. That was because when the onset is sudden the illness becomes severe. This result was different from what was noted in USA, where seizure onsets were not significant associated factor of the prevalence of ADHD.

Like studies conducted in Korea^8^, USA^14^ and India^17^ age in this work had no significant association with the magnitude of ADHD. The number of antiepileptic drugs, age at onset and prolonged antiepileptic treatments were not associated significantly with ADHD in the current study in distinction from the result of a study conducted in China^9^.

In contrast to the studies conducted in Korea^8^ and Tanzania^18^, the type of epilepsy syndrome had no significant association with the magnitude of ADHD in our study. The institution-based cross-sectional design of our study has limited its generalizability as well as its capacity of establishing temporal relationships.

## Conclusion and recommendation

The magnitude of ADHD among children with seizure disorders was relatively higher in this attempt than most study reports worldwide. Despite this high prevalence, only a few children were diagnosed as having ADHD. Male sex, sudden onsets of seizure, family history of seizure and that of other psychiatric disorders as well as uncontrolled seizures were factors that increased the odds of ADHD. To Increasing an early detection and management of co-morbid ADHD training should be given about mental health problems for health professionals working in pediatric seizure clinics. Treating seizure disorder appropriately and controlling seizure could minimize the risk of having co-morbid ADHD.
